# A pilot study of medical student attitudes to, and use of, commercial movies that address public health issues

**DOI:** 10.1186/1756-0500-4-111

**Published:** 2011-04-07

**Authors:** Peter Gallagher, Nick Wilson, Richard Edwards, Rachael Cowie, Michael G Baker

**Affiliations:** 1 Medical Education Unit, University of Otago, Wellington, PO Box 7343 Wellington South 6021, New Zealand; 2 Department of Public Health, University of Otago, Wellington, PO Box 7343 Wellington South 6021, New Zealand

## Abstract

**Background:**

An innovative approach to learning public health by using feature-length commercial movies was piloted in the fourth year of a medical degree. We aimed to explore how students responded to this approach and the relative effectiveness of two promotional strategies. Firstly we placed DVDs of 15 movies (with public health-related content) in the medical school library. Then alternating groups of students (total n = 82 students) were exposed to either a brief promotional intervention or a more intensive intervention involving a class presentation. The response rates were 99% at baseline and 85% at follow-up.

**Findings:**

The level and strength of support for using movies in public health training increased after exposure to the public health module with significantly more students "strongly agreeing". Student behaviour, in terms of movies viewed or accessed from the library, also suggested student interest. While there were no statistically significant differences in median viewing or library access rates between the two intervention groups, the distribution of viewing patterns was shifted favourably. Those exposed to the more intensive intervention (class presentation) were significantly more likely to have reported watching at least one movie (97% vs. 81%; p = 0.033) or to having accessed at least one movie from the library (100% vs. 70%, p = 0.0001).

**Conclusions:**

This pilot study found that the students had very positive attitudes towards viewing public health-related commercial movies. Movie access rates from the library were also favourable.

## Background

Popular feature-length commercial movies have long been recognised as a useful educational tool and have been used in the teaching of management ethics, sociology, psychology [1-5]. Such movies have also regularly been used as learning resource in the education of health-related professionals [4,6-10]. However, there has been little documented use of commercial movies in public health related education (i.e., we identified just one Medline-indexed study [11], which related to environmental health training).

The early use of visual media in educational settings were predominantly documentary or instructional in nature and were used to enhance or replace formal lectures, and to enable more students to gain insights into clinical activities previously only available to the few. In the last 20 years or so, celluloid film has been replaced by VHS and more recently DVD recordings. This has made it much easier to use feature-length commercial movies in the classroom [5]. Most commonly a movie or selected excerpts is shown with the tutor present and it is used to impart information and as a catalyst for class discussion. The choice of movie, the identification of the important learning points and the topics for discussion are invariably determined in advance by the teacher. Indeed, some educators describe precisely how to employ movies in the classroom: "Popular movies should not be used as a form of "entertainment" for students: it is preferable that the teacher attends the film's projection with his or her students" [12]. These are conventional approaches to the use of such visual media which do not maximise the flexibility and portability presented by VHS and DVD recordings.

Mindful of the practical and pedagogical challenges presented when attempting to be innovative, this pilot study explored the promotion of popular feature-length commercial movies during a five week long public health training module. In particular, we aimed to explore student attitudes and their viewing behaviour.

## Methods

### Participants

The participants were fourth year medical students (n = 82) undertaking a five week training module in public health in six groups during 2009 (Wellington, New Zealand). We excluded one student who had previously watched all the selected public health movies as part of the movie selection process (during a Summer Studentship).

### Movie selection

Fifteen feature-length commercial movies with public health themes and favourable review ratings were selected through a systematic process (see a description elsewhere [13], and on a website with a more detailed online report which includes detailed descriptions of the selected movies [14]). These movies were intended for the general public, initially screened at cinemas and subsequently widely available via DVD rental stores. They were all products of the entertainment industry and so the explicit or implicit public health themes were embedded in a product that was created primarily as a leisure activity. That is these movies were not primarily designed as educational vehicles for students in the health sciences or any other discipline. Amongst the public health themes were those of tobacco, nutrition, homelessness, prejudice, corporate decision making, environmental pollution, and health care systems (see Table 1 for movie titles and brief descriptions).

**Table 1:** Specific movie popularity based on self-reported viewing (at baseline and during the public health module) and DVD withdrawal data from the medical school library (for n = 82 students)

		**Self-report of having already seen the movie at baseline**		Self-report of having watched the movie during the module*		Library data on withdrawals*	
**Movie title (year)**	**Brief description (using the wording on the hand-out provided to all the students at the start of the module)**	**N**	**Row %**	**N**	**Row %**	**N**	**Row %**
*A Civil Action (1998)*	A drama about a law suit and toxic pollutants causing health problems (stars John Travolta).	1	1.2	7	8.5	13	15.9
*An Inconvenient Truth (2006)*	A documentary about climate change by Al Gore.	27	33.3	10	12.2	17	20.7
*And the Band Played On (1993)*	A drama about how the HIV/AIDS epidemic emerged in America and the response to it.	4	4.9	16	19.5	18	22.0
*Born into Brothels (2004)*	A documentary about the children of sex workers in an Indian city - and how a photographer helps them.	6	7.4	13	15.9	20	24.4
*Bowling for Columbine (2002)*	A quirky documentary about gun control in America (directed by Michael Moore).	36	44.4	9	11.0	7	8.5
*Bright Leaves (2003)*	A documentary about smoking, tobacco and personal history set in North Carolina.	1	1.2	2	2.4	6	7.3
*Dark Days (2000)*	A documentary about homeless people living in the New York subway - and how their housing problem is helped.	3	3.7	6	7.3	12	14.6
*Erin Brockovich (2000)*	A drama about a law suit and toxic pollutants causing health problems (stars Julia Roberts).	51	63.0	12	14.6	11	13.4
*Sicko (2007)*	A quirky documentary about access to health care in America compared to other countries (directed by Michael Moore).	30	37.0	27	32.9	25	30.5
*Super Size Me (2004)*	A quirky documentary about fast food and obesity (set in America).	48	59.3	21	25.6	15	18.3
*The Constant Gardener (2005)*	A drama about unethical corporate behaviour (pharmaceutical industry) set in Kenya.	31	38.3	16	19.5	20	24.4
*The Corporation (2003)*	A documentary about corporate behaviour, globalisation, and environmental health hazards.	7	8.6	8	9.8	11	13.4
*The Insider (1999)*	A drama based on a scientist who reveals information about tobacco industry practices (stars Russell Crowe).	15	18.5	10	12.2	20	24.4
*The Yes Men (2005)*	A humorous documentary about globalisation and corporate behaviour.	12	14.8	9	11.0	14	17.1
*Who Killed the Electric Car? (2006)*	A documentary (in murder mystery style) that examines why the electric car was withdrawn from California.	7	8.6	11	13.4	18	22.0
*Total*		279	-	177	-	227	-

Note: * Percentages are based on the denominator of the whole group (n = 82) with the assumption that those not completing the end-of-module questionnaire did not watch these movies (intention-to-treat analysis).

### Interventions

DVDs of the 15 movies were placed in the medical school library and were made available free of charge to the fourth year students. The first, third and fifth student group received a minimal intervention which involved a brief promotional presentation at the start of the module and a printed list of the movies with a short précis (with movie usage being optional and not assessed). The students were encouraged to view the movies away from the classroom setting (i.e., usually in their own homes) and encouraged to consider the nature of the public health messages contained within each movie. Thus the educational approach we adopted was one that incorporated an important concept in the context of student learning; that of constructivism [15]. This intervention was delivered to three groups of students (n = 40 students in total).

The second, fourth and sixth groups received the more intensive intervention which involved the minimal intervention plus the students being required to do a 5-minute class presentation on one of the 15 movies (their choice) at the end-of-the-module. This intervention was applied to three groups of students (n = 42 students in total). The class presentation was delivered to the other students (and a tutor) at a dedicated session in the last week of the module. Students were asked to summarise the movie, discuss the public health themes, and to say if they would recommend the movie to others. This session was also not formally assessed but there was an expectation that all students would participate as part of the public health module. The tutor at these sessions was one of the authors (PG), though other co-authors attended the session (as observers) on occasions.

We purposefully chose to deliver the interventions to alternate student groups during the year (rather than randomising interventions to groups) so as to ensure a more even distribution of the interventions throughout the year. This was because we anticipated progressive reduction in voluntary movie viewing by students as end-of-the-year exams approached and as other pressures mounted (e.g., to obtain jobs over the subsequent summer months and to earn money to pay mounting debts). Randomisation of interventions at the level of the student was not considered appropriate as the intervention was designed for group level delivery and involved group interaction (e.g., group discussion after each class presentation).

### Measurements

Immediately after the introductory session on day one of the public health module, and as part of best practice in the evaluation of educational innovation, all students completed an anonymous written questionnaire that collected baseline attitudinal and movie viewing data. Another similar written questionnaire was completed at the end-of-the-module. Free text fields were included in the questionnaires to obtain additional ideas and comments from the students. In addition to self-report data on movie usage, data on registered withdrawals of movies from the medical school library were also collected (to the end of the last module of 2009). As the withdrawal data was non-anonymised it was possible to link the names of the students with the intervention group they were in.

## Results

Nearly all the students (99%, n = 81/82) completed the baseline questionnaire and a large majority completed the end-of-module questionnaire i.e., 90% exposed to the minimal intervention (36/40) and 81.0% (34/42) exposed to the more intensive intervention (85% overall).

A slight majority of the students were women (56%). Twelve (15%) were students from the Middle East and South-East Asia who came to New Zealand to study medicine. Thirty-six (43%) stated that they currently shared a flat or house with another fourth year medical student/s. Of these 17 reported that one or more of the other students sharing their house of flat had completed a public health module earlier in the year.

The reported median frequency of viewing movies in general (i.e., at least 70 minutes long) on a DVD, television or at a movie theatre was "1-2 per month". However, 5% reported a level of 3+ per week and none reported a viewing frequency of less than one per six months. From the list of 15 public health-related movies (see Table 1); an average of 3.4 movies per student had previously been seen (range 0 - 10). There was no significant difference in the mean number of public health movies previously seen between students who shared a house with other fourth year medical students and did not (means of 3.1 vs 3.6 respectively). Nor between students who had a flatmate who had already completed the public health module and all other living situations (means of 3.2 vs 3.6 respectively).

### Changes in attitudes to movies

At baseline, most students (84.0%, 95%CI = 74.7% - 90.8%) "agreed" or "strongly agreed" that watching movies helps "medical students learn more about social and health issues" (Table 2). Similarly, most thought that providing a list of public health-related movies was a worthwhile part of their training (82.7%, 95%CI = 73.3% - 89.8%). At the end-of-the-module these respective figures had both risen (92.9%, 95%CI = 84.9% - 97.3%; and 95.7%, 95%CI = 88.8% - 98.9%). The increase in total agreement ("agreed" plus "strongly agreed") was only just statistically significant for providing the list of movies but the increase in the "strongly agree" proportion was significant for both (odds ratio [OR] = 3.16, 95%CI = 1.47 - 6.82; and OR = 2.64, 95%CI = 1.27 - 5.53 respectively). While these changes occurred in both groups there were no significant differences in responses according to intervention group.

**Table 2:** Student attitudes to movies with public health and social themes

**Response**	**Does watching movies help medical students learn more about social and health issues?**				**Is providing you with a list of public health related movies a worthwhile component of the public health module in fourth year?**			
	**Baseline**		End-of-module*		**Baseline**		End-of-module*	
	**N**	**%**	**N**	**%**	**N**	**%**	**N**	**%**
Strongly disagree	0	0.0	1	1.4	1	1.2	0	0.0
Disagree	0	0.0	1	1.4	1	1.2	1	1.4
Neither agree nor disagree	12	14.8	2	2.9	10	12.3	2	2.9
Agree	50	61.7	32	45.7	48	59.3	31	44.3
Strongly agree	18	22.2	33	47.1	19	23.5	36	51.5
Did not answer	1	1.2	1	1.4	2	2.5	0	0.0
**Total**	**81**	**100**	**70**	**100**	**81**	**100**	**70**	**100**

Note: *Just those answering the end-of-module questionnaire (i.e., 70/82 of the total sample).

### Response to the interventions on viewing behaviour

At baseline there were no significant differences in movie viewing patterns between the two groups (Table 3). Data from the end-of-the-module questionnaire and the movie DVD withdrawal data from the library indicated no statistically significant differences in median viewing or withdrawals by intervention group (Table 3). Nevertheless, those in the more intensive "class presentation" intervention group were significantly more likely to have reported watching at least one movie (97.1% vs. 80.6%; p = 0.033). This significant difference was also found for withdrawals from the library of at least one movie (100.0% vs. 70.0%, p = 0.0001). There were no significant differences in movie viewing or withdrawals by time of year, despite exams being held at the end of the year.

**Table 3:** Public health-related movie viewing and movie withdrawal data from the medical school library by the two interventions*

**Measure**	**Students in the 3 "minimal intervention" groups** **(n = 40 students)**	**Students in the 3 more intensive intervention "class presentation" groups** **(n = 42 students)**
***Self-reported viewing at baseline***		
Proportion viewing movies at the frequency of 1-2 per week or higher	50.0%	42.1%
Mean number of movies viewed from the list of 15 public health-related movies	3.1	3.8

***Self-reported viewing at end-of-module***		
Mean number watched during the module:		
- all students	2.4 (95/40)	2.0 (82/42)
- for those answering the questionnaire (n = 36 & n = 34)	2.6 (95/36)	2.4 (82/34)
Percentage watching at least one movie (for those answering the questionnaire)	80.6% (29/36)	97.1% (33/34)**

***DVD withdrawals from the library (covering all withdrawals up to the end of the last public health module of the year)***		
Self-reported withdrawals		
- total	86	70
- mean	2.2	1.7
- median	2	2
Registered withdrawals#		
- total	112	115
- mean	2.8	2.7
- median	2	3
- range	0 - 11	1 - 7
Nil DVDs withdrawn	12	0
Percentage withdrawing at least one DVD	70% (28/40) (95%CI = 54.6%-82.6%)	100% (42/42)*** (95%CI = 93.1%-100%)

Notes:

* Unless otherwise indicated all analyses assigned zero values for those not completing the end-of-module questionnaire (i.e., an intention-to-treat analysis).

** Statistically significantly higher compared to the minimal intervention group (p = 0.033, Fisher exact 1-tailed test).

*** Statistically significantly higher proportion of 1 or more withdrawn compared to the minimal intervention group (p = 0.0001)

# As reported on the library computer system and excluding repeat withdrawals by the same person.

During the five week public health module, students completing the questionnaire watched an average of 2.5 movies each. The withdrawal data included a small proportion of post-module withdrawals and gave slightly higher mean levels (2.8 movies per student).

The most viewed movie at baseline from the list of 15 public health-related movies was "Erin Brockovich" which 63% of students had previously seen (Table 1). This was followed by "Super Size Me" at 59%. The most popular of the self-reported movies watched (n = 177) was "Sicko" at 33%. Sicko was also the most popular (31%) of DVDs accessed from the library (out of n = 227).

### Logistical aspects of DVD provision

A few students reported problems with the format of the DVDs not working on the players they used. To address this we purchased another set of DVDs that would definitely work on devices using the DVD format for New Zealand (these were available for both of the last two modules for the two different interventions). No DVDs were damaged or lost during the study and the medical school library reported that the borrowing process ran smoothly

### Free text analysis

Quantitative data analysis was supported by the analysis of the free text responses primarily to the following question: *Do you have any other comments or ideas about the educational value of movie watching by medical students?*

In their pre-module comments the students considered that the opportunity to learn about public health issues from viewing movies was more appealing and less boring than lectures. This form of education was expected to be more fun and an enjoyable way of learning: *A more engaging story based approach than just sitting through lectures"*.

However, some students thought that merely being invited to view a movie was a too informal approach to their learning and suggested that academic staff should offer structured guidelines to enable students to maximise their learning: *Every movie watching should end up with a group discussion"*.

Following the module, the opportunity to view movies and at the same time learn about public health issues was almost unanimously regarded as a good idea:

"They [the movies] are a great source of education:

*"If watched not just for entertainment but analysed properly makes it very interesting"*.

Viewing movies enabled students to: *Retain more information than that gained from a lecture"*.

The experience was variously described as:

*"An awesome way to learn"*,

*"It [the movie] made me think" *or

*"A great source of education"*.

However, not all students actually watched a movie during the duration of the module. This was because some students had previously seen one of the movies, some could not find the time, some were unable to borrow the movie that they wanted to watch and some were unable to play the movie due to the incompatibility of the DVD and the DVD playback facilities.

## Discussion

### Main findings

This study found high (84%) baseline support by medical students for the use of movies in their public health training. Furthermore, the level and strength of support increased after exposure to the module with significantly more students "strongly agreeing" to this approach.

In terms of behaviour (movies viewed or accessed), there was further evidence for student interest in these movies with public health themes. While there were no statistically significant differences in median numbers of movies viewed or accessed between the two intervention groups, the distribution of viewing patterns was shifted favourably. That is those exposed to the more intensive intervention (class presentation) were significantly more likely to have reported watching at least one movie (97% vs. 81%) or to have accessed at least one movie from the library (100% vs. 70%).

Making these public health DVDs available via the medical school library appeared feasible and there was no attrition in terms of damage and loss. However for this study the DVDs were only promoted to the students doing the public health module and not to other medical school staff and students.

This study indicates that senior medical students are likely to behave in a manner supportive of constructivist approaches to education. That is students were able to take a key role in deciding what learning was important, how that learning was to occur, and were active participants in their own learning. Further, the relationship between learning experiences (in this case viewing the movie) and the learning that the students extracted from that experience is definitively personal. Therefore, the student, as the viewer, interprets whatsoever they wish and it is not for another person to tell them what is or what is not important in any particular text. It important to note that key public health issues were identified by students in the absence of any formal direction from the lecturers.

That there was no significant difference in the number of movies watched by the intervention and non-intervention groups may be related to the fact that these students are in the fourth year of a demanding degree programme and functioned as highly motivated and independent learners. Alternatively, the non-intervention groups may have perceived that they were somehow missing out on an important learning opportunity. Another explanation may be that movies are entertaining and pleasurable and as one student remarked: *As a student, I would suspect it is mainly because of the novelty of getting to do something enjoyable that is also classed as learning... is relatively rare in medical school*.

Whatever the student motivation, an area not specifically explored in this study; the success of the introduction of movies was reflected in the students' positive reception to them as a learning resource.

A final comment is made in respect of the synopsis of each movie offered by members of the intervention groups at the end of their module (at the class presentation). It was possible that feedback from students was based upon a movie that a student viewed before, during the module, or even before they began their medical degree. Thus the relationship between the ability to identify key public health issues and exposure to the movie was not explored.

### Study limitations

This study was relatively small (only n = 82 students) and so should only be considered a pilot study. Also the "more intensive" intervention was actually not that "intensive" in that class presentations by students were quite brief (around 5 minutes and without audiovisual components) and these were not an assessed part of the public health module.

Furthermore, we did not seek to formally evaluate the extent to which students cognitively processed the public health themes in the movies or integrated the themes with other aspects of their training. Nevertheless, at the class presentation our view is that there were signs of such processing and attempts at follow-up learning (e.g., getting out a DVD on a related topic).

We also did not capture various spill-over benefits of public health movie viewing to fifth year medical students and to other members of the public who share accommodation with four-year medical students. Also having the movie DVDs now in the medical school library may contribute to the learning by other staff and students.

It is important to point out that the primary function of movies is that of an art form produced for commercial entertainment. A secondary purpose may be to gain accolades from within and without the film industry. However, this study demonstrates that selected movies may also be acceptable to medical students as an  educational tool. Furthermore, in the course of discussions during the individual brief presentations some students suggested other movies they considered suitable additions to those made available for the duration of this study (e.g., "Thank you for Smoking" "The Last King of Scotland", and "Samson and Delilah").

### Implications for further research and medical student education

Given the favourable results from this pilot study in terms of student attitudes and movie viewing data, further research is warranted. This work has suggested that movie viewing can successfully occur outside of the crowded medical school curriculum — and hence may be an attractive strategy to increase student exposure to public health as part of medical student training.

Firstly a larger study could better assess how worthwhile a more intensive "class presentation" intervention is relative to the minimal promotion intervention. For this the "class presentation" could involve more formal components around teasing out the public health themes and relating these to what else the students have learnt during their public health module, and could be included in the summative assessment process for the module. Secondly the value of other promotional techniques could be explored. For example, the use of promotional signage within the medical school library to alert students to the movies could be considered. Library or medical school website based promotion could also be used. Running an occasional evening session with one of the movies could also be a way to highlight the existence of the movie collection.

## Competing interests

The authors declare that they have no competing interests.

## Authors' contributions

The study was conceived by NW and managed by PG, NW and RE. NW, RC and MB contributed to developmental work on movie selection. PG ran all the interactive sessions with the students and collated and analysed the student feedback. NW analysed the quantitative data. The first draft of the manuscript was prepared by PG and NW. All authors contributed to revised drafts and all authors read and approved the final manuscript.
